# Association between Body Mass Index and All-Cause Mortality in Hypertensive Adults: Results from the China Stroke Primary Prevention Trial (CSPPT)

**DOI:** 10.3390/nu8060384

**Published:** 2016-06-22

**Authors:** Wei Yang, Jian-Ping Li, Yan Zhang, Fang-Fang Fan, Xi-Ping Xu, Bin-Yan Wang, Xin Xu, Xian-Hui Qin, Hou-Xun Xing, Gen-Fu Tang, Zi-Yi Zhou, Dong-Feng Gu, Dong Zhao, Yong Huo

**Affiliations:** 1 Department of Cardiology, Peking University First Hospital, Beijing 100034, China; ywyy2008@163.com (W.Y.); lijianping@medmail.com.cn (J.-P.L.); drzhy1108@163.com (Y.Z.); fang9020@126.com (F.-F.F.); 2 National Clinical Research Center for Kidney Disease, State Key Laboratory for Organ Failure Research, Renal Division, Nanfang Hospital, Southern Medical University, Guangzhou 510515, China; xipingxu126@126.com (X.-P.X.); binyanwang126@126.com (B.-Y.W.); xux007@163.com (X.X.); xianhuiqin@126.com (X.-H.Q.); 3 Institute for Biomedicine, Anhui Medical University, Hefei 230032, China; ausachina@163.com (H.-X.X.); tanggenfu@163.com (G.-F.T.); zhouziyi19920319@126.com (Z.-Y.Z.); 4 State Key Laboratory of Cardiovascular Disease, Fuwai Hospital, National Center for Cardiovascular Diseases, Chinese Academy of Medical Sciences and Peking Union Medical College, Beijing 100037, China; gudongfeng@vip.sina.com; 5 Department of Epidemiology, Beijing Anzhen Hospital, Capital Medical University, Beijing Institute of Heart, Lung and Blood Vessel Diseases, Beijing 100029, China; deezhao@vip.sina.com

**Keywords:** obesity, body mass index, mortality, hypertension, China

## Abstract

The association between elevated body mass index (BMI) and risk of death has been reported in many studies. However, the association between BMI and all-cause mortality for hypertensive Chinese adults remains unclear. We conducted a post-hoc analysis using data from the China Stroke Primary Prevention Trial (CSPPT). Cox regression analysis was performed to determine the significance of the association of BMI with all-cause mortality. During a mean follow-up duration of 4.5 years, 622 deaths (3.0%) occurred among the 20,694 participants aged 45–75 years. A reversed J-shaped relationship was observed between BMI and all-cause mortality. The hazard ratios (HRs) for underweight (<18.5 kg/m^2^), overweight (24.0–27.9 kg/m^2^), and obesity (≥28.0 kg/m^2^) were calculated relative to normal weight (18.5–23.9 kg/m^2^). The summary HRs were 1.56 (95% CI, 1.11–2.18) for underweight, 0.78 (95% CI 0.64–0.95) for overweight and 0.64 (95% CI, 0.48–0.85) for obesity. In sex-age-specific analyses, participants over 60 years of age had optimal BMI in the obesity classification and the results were consistent in both males and females. Relative to normal weight, underweight was associated with significantly higher mortality. Excessive weight was not associated with increased risk of mortality. Chinese hypertensive adults had the lowest mortality in grade 1 obesity.

## 1. Introduction

Obesity is a global epidemic issue that is highly prevalent in both developed and developing countries; it affects people of both sexes and all ages, has adverse health consequences, accrues large economic costs, and has negative social implications [1]. Body mass index (BMI), defined as weight in kilograms divided by the square of the height in meters, is commonly used in clinical practice to screen for overweight and obesity and to guide weight loss recommendations. In China, an increasingly large proportion of the population has a BMI ≥ 25 kg/m^2^, the standard definition of overweight [2]. Although obesity has been demonstrated to be associated with multiple non-communicable diseases, including hypertension, type 2 diabetes, coronary heart disease, stroke and several cancers [3], the association between BMI and all-cause mortality remains controversial: a direct association, a J-shaped, a U-shaped, or a reversed J-shaped relationship have all been recently reported [4,5,6,7,8].

The World Health Organization (WHO) defines the following six categories based on BMI values: <18.5 kg/m^2^ = underweight; 18.5 to 24.9 kg/m^2^ = normal weight; 25.0 to 29.9 kg/m^2^ = overweight; 30.0 to 34.9 kg/m^2^ = grade 1 obesity; 35.0 to 39.9 kg/m^2^ = grade 2 obesity; and ≥40 kg/m^2^ = grade 3 obesity [9]. These definitions of overweight and obesity are mainly based on criteria derived from studies that involved populations of European origin. It has been suggested that the associations of BMI with body composition and health outcomes may differ between Asian and European populations [10]. The BMI cut-off points for overweight and obesity should be lower for Asian populations than they are for European populations (suggested cut-off points for Asians are ≥23.0 kg/m^2^ for overweight and ≥27.5 kg/m^2^ for obesity) [10]. However, a consensus statement from the WHO concluded that the available data were not sufficient to support Asian-specific cut-off points [10]. The Ministry of Health of the People’s Republic of China determined a reclassification of BMI for Chinese adults that differs from the WHO classification: underweight (<18.5 kg/m^2^), normal weight (18.5–23.9 kg/m^2^), overweight (24.0–27.9 kg/m^2^) and obesity (≥28.0 kg/m^2^) [11].

For decades, the prevalence of hypertension has been increasing in China, and by the year 2010 it reached 20% [12]. During the period of 2005–2009, about 42% of Chinese adults aged 35–70 years were hypertensive [12]. Although obesity is clearly regarded as a risk factor for developing hypertension, the optimal BMI for middle-aged or elderly hypertensive Chinese remains unclear. Therefore, the objective of this study was to evaluate the relationship between BMI and all-cause mortality in hypertensive Chinese adults.

## 2. Materials and Methods

### 2.1. Data Sources

This post-hoc analysis utilizes data from the China Stroke Primary Prevention Trial (CSPPT), which enrolled 20,702 subjects with primary hypertension in a multi-community, randomized, double-blind, controlled trial to assess whether enalapril maleate and folic acid supplementation was more effective in reducing risk of stroke than enalapril maleate supplementation alone. This study was conducted in accordance with the principles of the Declaration of Helsinki. The Human Subjects Committee at the Biomedical Institute of Anhui Medical University approved the study protocol. All patients provided written informed consent prior to data collection.

### 2.2. Participants and Treatment

The methods and primary results of the CSPPT trial have been reported elsewhere [13]. Briefly, the CSPPT was conducted from 19 May 2008 to 24 August 2013 in 32 communities in Jiangsu and Anhui Provinces in China. Eligible participants were men and women aged 45–75 years old who had hypertension, defined as seated resting systolic blood pressure (SBP) ≥140 mmHg or diastolic blood pressure (DBP) ≥90 mmHg at both the screening and recruitment visit, or who were on anti-hypertensive medication. The major exclusion criteria included history of physician-diagnosed stroke, myocardial infarction (MI), heart failure, post-coronary revascularization, or congenital heart disease.

The current analysis was designed to investigate the relationship between BMI and all-cause mortality in this cohort. All-cause mortality included death due to any reason. After excluding eight subjects with missing information on weight and height, the final analysis included 20,694 subjects. Participants contributed person-years from the date of recruitment until date of death or end of follow-up (24 August 2013). BMI classifications as set according to the guidelines from the Ministry of Health of the People’s Republic of China were used and included: underweight (<18.5 kg/m^2^), normal weight (18.5–23.9 kg/m^2^), overweight (24.0–27.9 kg/m^2^) and obesity (≥28.0 kg/m^2^). At the initial study visit trained research staff measured and recorded height (to the nearest 0.1 cm) and weight (to the nearest 0.1 kg) for each participant. In addition, trained staff collected baseline demographic data, medical history, and medication use. Cigarette smoking was classified into never, former, and current smoker (defined as smoke at least one cigarette per day for more than one year). Alcohol drinking was stratified into never, former and current drinker (defined as drink alcohol at least twice weekly for more than one year). Education was categorized into illiterate (0 years of education), primary school (1–6 years), and secondary school (>6 years) or above. Stress was defined as mild, moderate or severe according to the participant’s personal evaluation.

### 2.3. Follow-Up and Outcomes

Patients were evaluated every three months for an average of five years to assess blood pressure (BP), adherence to medication, and adverse outcomes including stroke, composite major cardiovascular (CV) events and resultant death, and all-cause death. The study outcomes were adjudicated according to standard criteria by a clinical end points committee.

### 2.4. Statistical Analysis

All participants were divided into four groups according to the Chinese classification for BMI (<18.5, 18.5–23.9, 24.0–27.9, ≥28.0 kg/m^2^). Baseline characteristics of all participants were compared using the analysis of variance (ANOVA) for continuous variables and the χ^2^ test for categorical variables. All-cause mortality was first assessed using the Kalpan-Meier method and log-rank tests, and then multivariable Cox proportional-hazards regression models were applied to calculate hazard ratios (HR) and 95% confidence intervals (CI) for the risk of all-cause mortality in each of the BMI groups. Potential confounders were adjusted including sex, age, center, baseline and on-treatment BP, smoking status, alcohol drinking, education, stress, fasting blood glucose (FBG), total cholesterol (TC), triglycerides (TG), serum creatinine (SCr), homocysteine (Hcy) and albumin. Further stratified analyses by subgroups including sex, age, center, smoking and alcohol drinking status, education, stress and albumin levels were also explored by Cox proportional-hazards regression models to test for consistency of results. All tests were two-sided, and *p*-values less than 0.05 were considered statistically significant. All analyses were performed by EmpowerStats [14] and the statistical package R [15].

## 3. Results

### 3.1. Patient Characteristics

Baseline characteristics of all patients are presented in Table 1. Of the 20,694 participants, 59.0% were female, and the mean age was 60.0 years (SD, 7.5 years), with a range from 45 to 75 years. The mean BMI was 25.0 kg/m^2^ (SD, 3.4 kg/m^2^). Men had a lower of BMI 24.2 kg/m^2^ (SD, 3.7 kg/m^2^) than women, who had a BMI of 25.4 kg/m^2^ (SD, 3.4 kg/m^2^). The percentages of underweight, normal weight, overweight and obese were 2.5%, 39.1%, 38.9% and 19.5%, respectively. Higher BMI categories were associated with younger age, female gender, better education, and higher levels of FBG, TC, TG, albumin, and baseline and on-treatment blood pressure measurements. Lower BMI categories were associated with higher HDL-C levels and current smoking status.

### 3.2. BMI and Mortality

The relationship between BMI and overall mortality for all patients with hypertension is shown in Table 2. During a median follow-up period of 4.5 years (88,466.64 person-years), 622 deaths occurred. Specifically, with increasing BMI, the all-cause mortality rate in underweight, normal weight, overweight and obese groups was 7.8%, 3.9%, 2.4% and 1.8%, respectively. According to the BMI category, the all-cause mortality rate per 1000 person-years was 17.63, 8.93, 5.76 and 4.24, respectively. In a crude Cox proportional-hazards regression model, the HRs were 1.97 (95% CI, 1.42–2.73) for underweight, 0.64 (95% CI, 0.54–0.77) for overweight and 0.47 (95% CI, 0.37–0.61) for obesity compared with normal weight. In model II, after adjusting for sex, age, center, baseline and on-treatment BP, smoking status, alcohol drinking, education, stress and serum biochemical measurements, the HRs were 1.56 (95% CI, 1.11–2.18) for underweight, 0.78 (95% CI, 0.64–0.95) for overweight and 0.64 (95% CI, 0.48–0.85) for obesity compared with normal weight.

**Figure 1:**
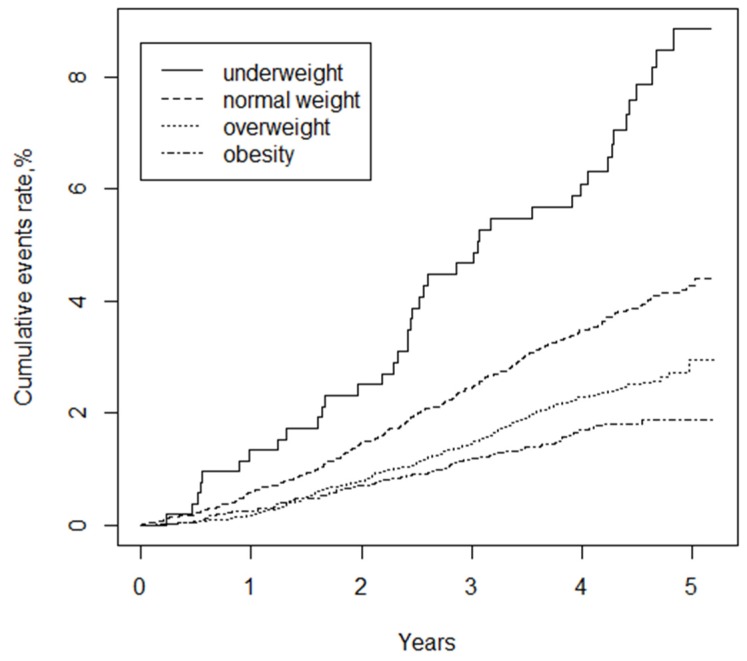
Kaplan-Meier curves of cumulative hazards of all-cause mortality stratified by BMI categories (underweight <18.5, normal weight 18.5–23.9, overweight 24.0–27.9, obesity ≥28.0 kg/m^2^). log-rank test, *p* < 0.001.

Figure 1 shows the Kaplan-Meier curves of the cumulative hazards of all-cause mortality stratified by BMI categories. All-cause mortality between each of the four BMI groups was significantly different (log-rank test, *p* < 0.001). With increased BMI, the cumulative mortality risk gradually decreased, rendering the underweight group with the maximum mortality risk.

Further stratified analyses were performed by important covariables including gender, center, smoking status, alcohol drinking status, education, stress and albumin levels (Table 3). There were no significant interactions in any of the subgroups (*p* > 0.05 for all comparisons). The beneficial effect appeared to be more pronounced in participants older than 60 years.

**Figure 2:**
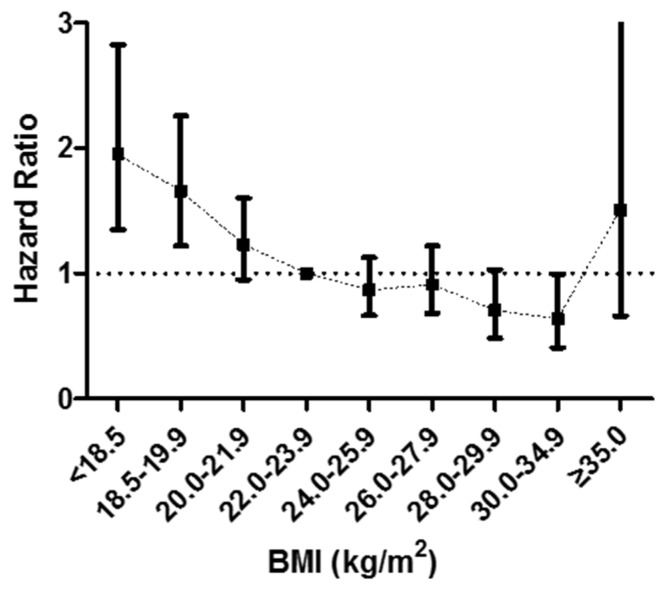
Hazard ratios for all-cause mortality according to narrower BMI categories. A BMI (in kg/m^2^) of 22.0–23.9 was used as the reference to estimate all HRs. Adjustment for sex, age, center, baseline and on-treatment BP, smoking status, alcohol drinking, education, stress, fasting blood glucose, total cholesterol, triglycerides, serum creatinine, homocysteine, albumin.

We subsequently repeated the analysis using narrower BMI categories (<18.5, 18.5–19.9, 20.0–21.9, 22.0–23.9, 24.0–25.9, 26.0–27.9, 28.0–29.9, 30–34.9, ≥35 kg/m^2^) to delineate the relationship between BMI and mortality with greater precision. Compared to patients with normal BMI (between 22.0 and 23.9 kg/m^2^), patients with grade 1 obesity (30–34.9 kg/m^2^) had the lowest risk of all-cause mortality (Figure 2). However, all-cause mortality increased drastically for patients with severe obesity (≥35 kg/m^2^).

## 4. Discussion

Our post-hoc analysis of this Chinese population with primary hypertension in the CSPPT showed the association between BMI and all-cause mortality followed a reversed J-shaped curve with increased risk at low and high BMI status. Even after adjusting for potential confounders, the results were similar. Compared to normal weight, the HRs were 1.56 (95% CI, 1.11–2.18) for underweight, 0.78 (95% CI, 0.64–0.95) for overweight and 0.64 (95% CI, 0.48, 0.85) for obesity. In this population, the optimal BMI was grade 1 obesity (30–34.9 kg/m^2^). Stratified analyses were consistent in all important covariables. Moreover, overweight or obese patients older than 60 years showed the most benefit.

Many studies conducted on different populations showed a U-shaped or J-shaped association between BMI and all-cause mortality, which is consistent with our results when applied to the underweight participants only [4,5,6,7,8]. In these studies, the population age distribution and whether subgroup analyses were performed by age group might be two major reasons for the controversial results. Janssen et al. found that BMI was inversely associated with mortality risk in elderly Asians [16]. Compared to middle-aged adults, some previous studies found that the optimal BMI among the elderly shifted upwards [17,18]. This finding is similar to what has been observed in other Asian regions such as Taiwan [19], Hong Kong [20], India and Bangladesh [8], among others [4,5,7]. Many of the elderly participants from these studies were residents of live-in care facilities, and/or were diagnosed with a health ailment such as hypertension, or were from undeveloped communities compared with urban residents. However, the optimal range of BMI proposed among the elderly varies among studies. As the world’s population ages and the number of hypertensive adults rises, our findings are important for health care professionals and policy-makers whose decisions affect elderly hypertensives.

Several potential explanations for the observed obesity paradox seen in our study exist. Firstly, reverse causality is a major problem in observational studies. For example, weight loss is classically experienced prior to death among the elderly who are ill. Secondly, socioeconomic status could confound the association between BMI and the risk of death, as people from less well-developed countries with a high BMI are more likely to have a higher socioeconomic status, allowing them better access to health care than those with a lower BMI [8]. However, the participants in our study are from rural communities, and a similar relation between BMI and socioeconomic status exists for them. Thirdly, in a meta-analysis of patients with existing coronary artery disease, those classified as overweight and obese had longer survival rates, potentially because of the benefit from the body’s reservation of nutrition within the population of elderly suffering from illness [21]. Fourthly, intensive pharmacotherapy could play a large role in the reverse J-shaped relationship. It has been demonstrated that patients with intensive pharmacotherapy are more likely to reach targets for secondary prevention. In population studies conducted in the USA and Canada, obese patients are more likely to meet targets for BP, lipids, and glycemic control than normal-weight patients [22]. In addition, hypertensive participants are relatively less healthy compared to the general population, although in our study we excluded patients with stroke and MI. Mary et al. found that among elderly Chinese with poor health status, there was an inverse, dose-response relationship between BMI and mortality, and this relationship was more pronounced in the group with the most morbidity compared to subjects of normal weight [23]. Another interpretation is that BMI can be a marker of adiposity, fitness and muscle mass, so that BMI maintenance in older people serves as an overall marker of health. Song et al. accessed the sex-age-specific association between BMI and all-cause mortality in the general population, and showed that the optimal BMI increased according to age and the trend was consistent in both males and females [24]. Similarly, Woo et al. found that in older elderly (aged over 75 years), a higher BMI was a protection from mortality [17].

Our study also has several limitations. Firstly, our participants were all middle-aged or elderly hypertensives; therefore, the result of this study cannot be generalized to all populations. Secondly, with the strict exclusion of the history of cardiovascular disease, cardiovascular cause of death counted for only 14% of all-cause death, which is less than other studies conducted on Chinese hypertensive adults. The association between BMI and a specific cause of death cannot be analyzed. Thirdly, in the current study, relatively few elderly were ≥30 kg/m^2^ (10% of total subjects) and ≥35 kg/m^2^ (1%); thus, the impact of obesity cannot be adequately assessed. Moreover, although we have excluded subjects with a baseline diagnosis of coronary heart disease, stroke, cancer, or acquired organic diseases, some preexisting illness may modify the association between BMI and mortality risk. However, according to our investigation, after excluding those subjects with no history of disease at baseline and who survived the first two years of follow-up, the BMI and all-cause mortality association was consistent with all 4.5 years of this study.

## 5. Conclusions

In conclusion, this study revealed a reversed J-shaped association between BMI and all-cause mortality in Chinese hypertensive adults. Relative to normal weight, underweight was associated with significantly higher mortality. Excessive weight was not associated with increased risk of mortality. A BMI classification of grade 1 obesity for Chinese hypertensive adults without stroke or MI showed the lowest mortality. Additional large-scale prospective studies with well-controlled confounding factors are needed to further address this important public health issue.

## Figures and Tables

**Table 1:** Baseline characteristics of the study participants by BMI categories ^1^.

Variables	BMI Categories, kg/m^2^					
	All Subjects	<18.5	18.5–23.9	24.0–27.9	≥28.0	*p*-Value
Number (%)	20,694	526(2.5)	8083 (39.1)	8043 (38.9)	4042 (19.5)	
Age, mean (SD), years	60.0 (7.5)	64.6 (6.7)	61.3 (7.4)	59.3 (7.5)	58.2 (7.4)	<0.001
BMI, mean (SD), kg/m^2^	25.0 (3.7)	17.5 (0.8)	21.8 (1.4)	25.8 (1.1)	30.4 (2.2)	<0.001
Male, No. (%)	8491 (41.0)	271 (51.5)	3863 (47.8)	3137 (39.0)	1220 (30.2)	<0.001
Center, No. (%)						<0.001
Anqing	5211 (25.2)	350 (66.5)	3119 (38.6)	1406 (17.5)	336 ( 8.3)	
Lianyungang	15,483 (74.8)	176 (33.5)	4964 (61.4)	6637 (82.5)	3706 (91.7)	
SBP, mean (SD), mmHg						
baseline	166.9 (20.4)	164.3 (17.9)	166.0 (20.0)	167.1 (20.4)	168.4 (21.4)	<0.001
on-treatment	139.4 (10.9)	138.8 (10.8)	138.9 (11.0)	139.4 (10.8)	140.4 (11.0)	<0.001
DBP, mean (SD),mmHg						
baseline	94.1 (11.9)	88.0 (11.4)	91.8 (11.7)	95.0 (11.6)	97.7 (11.9)	<0.001
on-treatment	82.9 (7.5)	78.9 (7.7)	81.5 (7.4)	83.5 (7.2)	85.3 (7.2)	<0.001
Pulse, mean (SD),bpm	73.6 (10.1)	74.0 (11.0)	73.6 (10.2)	73.5 (10.1)	73.8 (9.9)	0.53
Smoking status (%)						<0.001
never	14,252 (68.9)	293 (55.7)	5015 (62.1)	5791 (72.0)	3153 (78.0)	
former	1567 (7.6)	35 (6.7)	604 (7.5)	649 (8.1)	279 (6.9)	
current	4867 (23.5)	198 (37.6)	2459 (30.4)	1601 (19.9)	609 (15.1)	
Alcohol drinking (%)						<0.001
never	14,265 (69.0)	343 (65.2)	5155 (63.8)	5665 (70.5)	3102 (76.8)	
former	1458 (7.0)	52 (9.9)	609 (7.5)	540 (6.7)	257 (6.4)	
current	4960 (24.0)	131 (24.9)	2313 (28.6)	1836 (22.8)	680 (16.8)	
Education (%)						<0.001
illiterate	13,221 (63.9)	365 (69.4)	5261 (65.1)	4999 (62.2)	2596 (64.3)	
primary	3446 (16.7)	103 (19.6)	1468 (18.2)	1296 (16.1)	579 (14.3)	
secondary or above	4015 (19.4)	58 (11.0)	1347 (16.7)	1745 (21.7)	865 (21.4)	
Stress (%)						0.035
mild	12,578 (60.8)	289 (54.90)	4846 (60.0)	4960 (61.7)	2479 (61.4)	
moderate	6921 (33.4)	204 (38.8)	2765 (34.2)	2627 (32.7)	1323 (32.8)	
severe	1189 (5.7)	33 (6.3)	464 (5.7)	454 (5.6)	237 (5.9)	
FPG, mean (SD), mmol/L	5.8 (1.7)	5.3 (1.6)	5.6 (1.6)	5.9 (1.8)	6.1 (1.7)	<0.001
TC, mean (SD), mmol/L	5.5 (1.2)	5.0 (1.2)	5.4 (1.2)	5.6 (1.2)	5.7 (1.2)	<0.001
TG, mean (SD), mmol/L	1.7 (1.2)	1.2 (0.5)	1.4 (0.8)	1.8 (1.0)	2.0 (1.8)	<0.001
HDL-C, mean (SD), mmol/L	1.3 (0.4)	1.6 (0.4)	1.5 (0.4)	1.3 (0.3)	1.2 (0.3)	<0.001
SCr, mean (SD), umol/L	66.0 (19.3)	67.6 (16.7)	67.3 (19.6)	65.6 (20.5)	64.0 (16.3)	<0.001
Hcy, mean (SD), umol/L	14.5(8.4)	14.3 (7.7)	14.5 (8.1)	14.5 (8.4)	14.3 (8.9)	0.534
Albumin, mean (SD), g/L	49.0 (5.9)	47.57 (6.6)	48.57 (6.0)	49.34 (5.7)	49.34 (5.6)	<0.001
Treatment (%)						0.832
Enalapril	10,352 (50.0)	271 (51.5)	4053 (50.1)	4025 (50.0)	2003 (49.6)	
Enalapril-Folic Acid	10,342 (50.0)	255 (48.5)	4030 (49.9)	4018 (50.0)	2039 (50.4)	

^1^ For the determination of *p*-values, two-sample *t*-tests for continuous variables, chi-square tests for categorical variables. SBP, systolic blood pressure; DBP, diastolic blood pressure; FBG, fasting blood glucose; TC, total cholesterol; TG, triglycerides; SCr, serum creatinine; Hcy, homocysteine.

**Table 2:** Hazard ratios for all-cause mortality according to BMI status ^1^.

BMI kg/m^2^	*N*	Event (%)	Curde Rate	All-Cause Mortality			
				Model 1	*p-*Value	Model 2	*p-*Value
Continuous	20,694	622 (3.0%)	7.03	0.90 (0.88, 0.92)	<0.001	0.94 (0.91, 0.96)	<0.001
<18.5	526	41 (7.8%)	17.63	1.97 (1.42, 2.73)	<0.001	1.56 (1.11, 2.18)	0.010
18.5–23.9	8083	312 (3.9%)	8.93	1		1	
24.0–27.9	8043	197 (2.4%)	5.76	0.64 (0.54, 0.77)	<0.001	0.78 (0.64, 0.95)	0.012
≥28	4042	72 (1.8%)	4.24	0.47 (0.37, 0.61)	<0.001	0.64 (0.48, 0.85)	0.002

^1^ Multivariable Cox proportional-hazards regression models were applied to calculate hazard ratios (HR) and 95% confidence intervals (CI) for the risk of all-cause mortality in each of the BMI groups. A BMI (kg/m^2^) of 18.5–23.9 was used as the reference to estimate all HRs. Crude rates are all-cause mortality per 1000 person-years. Model 1: crude; Model 2: adjustment for sex, age, center, baseline and on-treatment BP, smoking status, alcohol drinking, education, stress, fasting blood glucose, total cholesterol, triglycerides, serum creatinine, homocysteine, albumin.

**Table 3:** Multivariable hazard ratios for mortality ^1^.

Variables	*N*	Death, *n*	%	BMI Categories( kg/m^2^)							Adjusted * *p* for Interaction
				<18.5		18.5–23.9	24.0–27.9		≥28		
				HR 95%CI	*p-*Value	HR 95%CI	HR 95%CI	*p-*Value	HR 95%CI	*p-*Value	
**All Participants**	20,694	622	3.0	1.56 (1.10, 2.14)	0.012	1	0.80 (0.66, 0.97)	0.026	0.66 (0.49, 0.87)	0.003	
**Sex**											0.692
Females	12,203	258	2.1	1.24 (0.66, 2.33)	0.505	1	0.78 (0.58, 1.05)	0.097	0.60 (0.41, 0.89)	0.010	
Males	8491	364	4.3	1.74 (1.16, 2.58)	0.007	1	0.82 (0.63, 1.07)	0.144	0.71 (0.46, 1.09)	0.119	
**Center**											0.719
Anqing	5211	218	4.2	1.58 (1.04, 2.38)	0.030	1	0.72 (0.49, 1.05)	0.090	0.88 (0.45, 1.72)	0.709	
Lianyungang	15,483	404	2.6	1.59 (0.88, 2.88)	0.125	1	0.84 (0.67, 1.06)	0.153	0.65 (0.47, 0.90)	0.009	
**Age**											0.588
<60	10,469	172	1.6	1.82 (0.78, 4.25)	0.167	1	0.82 (0.56, 1.19)	0.302	0.84 (0.52, 1.34)	0.463	
≥60	10,225	450	4.4	1.62 (1.13, 2.34)	0.009	1	0.75 (0.60, 0.95)	0.016	0.53 (0.36, 0.76)	<0.001	
**Smoking Status**											0.298
Never	14,254	327	2.3	1.40 (0.83, 2.37)	0.208	1	0.80 (0.61, 1.04)	0.090	0.62 (0.43, 0.89)	0.009	
Former	1570	75	4.8	3.76 (1.64, 8.67)	0.002	1	1.37 (0.75, 2.49)	0.305	1.28 (0.56, 2.90)	0.558	
Current	4869	219	4.5	1.33 (0.79, 2.25)	0.281	1	0.70 (0.50, 1.00)	0.052	0.60 (0.33, 1.09)	0.095	
**Alcohol**											
Never	14,265	355	2.5	1.54 (0.98, 2.43)	0.064	1	0.75 (0.58, 0.97)	0.026	0.63 (0.44, 0.89)	0.009	0.906
Former	1458	80	5.5	2.51 (1.17, 5.42)	0.019	1	0.85 (0.48, 1.48)	0.559	0.50 (0.20, 1.28)	0.148	
Current	4960	186	3.8	1.27 (0.65, 2.47)	0.482	1	0.93 (0.65, 1.34)	0.695	0.84 (0.46, 1.55)	0.581	
**Education**											0.599
Illiterate	13,221	411	3.1	1.43 (0.95, 2.15)	0.087	1	0.75 (0.59, 0.96)	0.021	0.67 (0.48, 0.94)	0.022	
Primary	3446	116	3.3	1.52 (0.70, 3.31)	0.290	1	0.98 (0.63, 1.53)	0.935	0.49 (0.22, 1.09)	0.080	
Secondary or above	4015	94	2.3	2.18 (0.88, 5.40)	0.093	1	0.78 (0.47, 1.29)	0.330	0.68 (0.33, 1.39)	0.290	
**Stress**											0.506
Mild	12,574	391	3.1	1.26 (0.79, 2.00)	0.333	1	0.73 (0.57, 0.94)	0.013	0.66 (0.47, 0.94)	0.019	
Moderate	6919	202	2.9	1.89 (1.13, 3.17)	0.016	1	0.91 (0.64, 1.29)	0.602	0.58 (0.33, 1.01)	0.054	
Severe	1188	28	2.4	2.97 (0.59, 14.93)	0.187	1	1.29 (0.49, 3.42)	0.604	0.90 (0.24, 3.32)	0.869	
**Albumin, g/L**											0.527
<49	11,813	412	3.5	1.48 (1.00, 2.18)	0.051	1	0.73 (0.57, 0.93)	0.012	0.68 (0.48, 0.97)	0.033	
≥49	8876	210	2.4	1.87 (0.98, 3.56)	0.058	1	0.87 (0.63, 1.20)	0.394	0.56 (0.34, 0.90)	0.016	

^1^ Cox proportional-hazards regression models to test interaction terms between BMI and other factors, but none were found to be significant. * Adjustment for sex, age, center, baseline and on-treatment BP, smoking status, alcohol drinking, education, stress, fasting blood glucose, total cholesterol, triglycerides, serum creatinine, homocysteine, albumin; sex, center, age, smoking status, alcohol, education, stress and albumin were not adjusted for in each corresponding subgroup analyses.

## Abbreviations

The following abbreviations are used in this manuscript:

BMI	body mass index
BP	blood pressure
CI	confidence interval
CSPPT	China Stroke Primary Prevention Trial
CV	major cardiovascular
DBP	diastolic blood pressure
FBG	fasting blood glucose
Hcy	homocysteine
HR	hazard ratio
MI	myocardial infarction
SBP	systolic blood pressure
SCr	serum creatinine
TC	total cholesterol
TG	triglycerides
WHO	The World Health Organization
